# The international ENIGMA-II substudy on postoperative cognitive disorders (ISEP)

**DOI:** 10.1038/s41598-021-91014-8

**Published:** 2021-06-02

**Authors:** Guy Haller, Matthew T. V. Chan, Christophe Combescure, Ursula Lopez, Isabelle Pichon, Marc Licker, Roxane Fournier, Paul Myles

**Affiliations:** 1 Division of Anesthesiology, Department of Acute Care Medicine, Geneva University Hospitals and Faculty of Medicine, University of Geneva, 4, Rue Perret-Gentil, 1211 Genève 14, Switzerland; 2 Department of Anaesthesia and Intensive Care, The Chinese University of Hong Kong, Prince of Wales Hospital, Shatin, New Territories, Hong Kong Special Administrative Region China; 3 Health Services Management and Research Unit, Department of Epidemiology and Preventive Medicine, Monash University, Melbourne, VIC Australia; 4 Division of Clinical Epidemiology, Department of Health and Community Medicine, University Hospitals of Geneva and Faculty of Medicine, University of Geneva, Geneva, Switzerland; 5 Unit of Neuropsychology and Logopedics, Department of Medicine, Cantonal Hospital of Fribourg, Fribourg, Switzerland; 6 Department of Anesthesiology and Perioperative Medicine, Alfred Hospital and Monash University, Melbourne, VIC Australia

**Keywords:** Health care, Medical research, Risk factors

## Abstract

There is a large controversy as to whether nitrous oxide (N_2_O) added to the anaesthetic gas mixture is harmful or harmless for postoperative cognitive function recovery. We performed a nested study in the ENIGMA-II trial and compared postoperative neurocognitive recovery of patients randomly receiving N_2_O (70%) or Air (70%) in 30% O_2_ during anesthesia. We included adults having non cardiac surgery. We compared recovery scores for episodic memory, decision making/processing speed and executive functions measured with the computerised Cambridge Neuropsychological Test Automated Battery (CANTAB). Assessments were performed at baseline, seven and ninety days. At first interim analysis, following recruitment of 140 participants, the trial was suspended. We found that the mean (95%CI) changes of scores for episodic memory were in the Pocock futility boundaries. Decision making/processing speed did not differ either between groups (P > 0.182). But for executive functions at seven days, the mean number (95% CI) of problems successfully solved and the number of correct box choices made was higher in the N2O group, P = 0.029. N_2_O with the limitations of an interim analysis appears to have no harmful effect on cognitive functions (memory/processing speed). It may improve the early recovery process of executive functions. This preliminary finding warrants further investigations.

## Introduction

The number of surgical procedures performed worldwide is estimated at 312 million^1^. Nitrous oxide (N_2_O) is used in a significant number of procedures. In the USA, for example, around 35% of all general anesthesia include nitrous oxide^2^. N_2_O is also increasingly administered with oxygen (50%) for pain and stress management in obstetrics during delivery, in pediatrics and ambulatory medicine for peripheral minor surgery. For general anesthesia, because of its weak anesthetic properties, it is used as an adjuvant that enables dose reduction of other anesthetic drugs and limits their side-effects. Considered for decades as innocuous, there is emerging evidence that N_2_O carries a number of potential side effects^3^. It enlarges natural air spaces (bowels, lungs, tympanic cavity). It can cause transitory leucopenia^4^, postoperative nausea and vomiting^5^. N_2_O also increases plasma homocysteine for up to a week after surgery^6,7^. Elevation of plasma homocysteine causes endothelial dysfunction and mismatches between cerebral metabolism and blood flow^8,9^. As a result N_2_O may also lead to cerebrovascular dysfunction resulting in delirium, delayed neurocognitive recovery or persisting neuro cognitive disorders^3,10^. Existing evidence on the true impact of N_2_O on postoperative cognitive performance and recovery is however controversial. Whilst some reports (both animal and human studies) attribute postoperative learning difficulties, loss of memory, disorientation^11–13^ and reduced psychomotor performance to N_2_O^14,15^, others including randomized trials, fail to identify any detrimental effect of N_2_O on cognitive performance^16–20^. Some authors even find in animal studies a neuroprotective effect of N_2_O^21^.

Thus, the role of N_2_O in the development of delayed neurocognitive recovery remains to be determined. As part of the ENIGMA-II randomized multicenter trial initially designed to assess cardiovascular complications in patients undergoing major non-cardiac surgery, we performed a nested study assessing postoperative neurocognitive recovery. Using three computerized neuropsychological tests (thirteen outcome scores) of the Cambridge Neuropsychological Test Automated Battery (CANTAB), we compared postoperative neurocognitive recovery of patients receiving N_2_O in the anesthetic gas mixture administered during surgery with patients receiving N_2_O free anesthesia.

## Methods

### Study design and participants

The International ENIGMA-II study on Postoperative Cognitive Disorders (ISEP) trial was a randomized, controlled, multicenter parallel-group study performed to assess the effect of N_2_O on postoperative neurocognitive recovery. It was a study nested in the original ENIGMA-II trial in two participating centers (Hong-Kong and Geneva), both University affiliated Hospitals. Adults aged at least 45 years, at risk of cardiovascular complications and having general anesthesia for non-cardiac surgery exceeding 2 h were eligible. We excluded patients with untreated deficit in Vitamin B6, B12 and folic acid, those with marked impairment of gas-exchange requiring inspiratory oxygen concentration > 0.5, those with specific circumstances where N_2_O is contraindicated (e.g. colorectal, thoracic surgery). In addition, we also excluded patients with mini mental state examination (MMSE) test score ≤ 24 and advanced Parkinson’s disease, those suffering from alcohol dependency or taking tricyclic antidepressants or neuroleptics. Patients with a handicap (i.e. visual impairment) likely to hinder the correct performance of the CANTAB computerized neuropsychological tests were also excluded. The study protocol was approved by the Central Ethics Committee of the Geneva University Hospitals CER: 08–075 (NAC 08–021) and Joint Chinese University of Hong Kong – New Territories East Cluster Clinical Research Ethics Committee (CRE-2012.197-T). It also received approval by the Swiss Agency for Therapeutic Products (Swiss Medics). The research project was performed in accordance with institutional standards and regulations. The study is registered with ClinicalTrials.gov, number NCT00430989 (reg.02.02.2007) and NCT02489097 (reg. 02.07.2015).

### Randomization, treatment allocation, blinding and data collection

The study was nested in the ENIGMA-II trial^22^ but was extended beyond the end of the original trial until May 2016. Following written informed consent from all study participants, they were randomized to receive a general anesthetic with a mixture of either N_2_O (70%) or Air (70%) in 30% O_2_. We used computer generated randomization in permuted blocks of 10 patients and stratified randomization by site. Group allocation could be accessed via an automated telephone voice-recognition service or in case of malfunction, it was sent directly by email to research staff. Patients, surgical team members, research staff including postoperative interviewers and cognitive testing assistants were blinded to group allocation. Only the anesthesiologist in charge was aware of the gas mixture provided during anesthesia.

On the day before surgery, after an initial screening for pre-existing cognitive disorders (MMSE test ≤ 24), Parkinson’s disease, visual or auditive impairment and other study exclusion criteria, a specially trained research assistant and a certified neuropsychologist performed the initial battery of cognitive tests CANTAB. The overall computerized battery includes 7 different categories of tests assessing the different domains of cognitive function (attention, executive functions, memory, visuospatial processing functions and language). These tests are designed to detect subtle changes in cognition. The CANTAB testing battery was preferred over other type of cognitive tests, since it is language-independent, culturally neutral and has been validated for the diagnosis of a wide range of cognitive disorders and syndromes^23^. For the study, we initially selected five tests to be performed at 7 and 90 days: screening tests MOT (Motor Screening Task), visual memory tests PAL(Paired Associates Learning), episodic memory tests PRM (Pattern Recognition Memory), decision making and processing speed tests RTI (Reaction time) and executive function tests OTS (One Touch Stocking of Cambridge). All these tests, except the MOT test, were chosen because they are assessing cognitive domains most likely to be influenced by anesthesia^24^. The MOT test is a standard screening test that is systematically performed before all other CANTAB tests in order to train participants to the use of the computer and touch screen technology^24^. It was therefore not selected as a main study outcome. The PAL test proved to be particularly challenging for patients in addition to all the other tests (average duration of testing sessions without PAL test: 45 min) and it was finally removed from the testing battery. Thus only the PRM test was used to assess memory, a choice which was approved by the neuropsychologists of our research team. Details of the CANTAB tests are provided in “Supplementary 1: Appendix 1”.

On the day of surgery, patients were randomly allocated (1:1) to N_2_O or N_2_O-free anesthesia. Group allocation prepared by study coordinators on each study site was provided in opaque sealed envelopes to the anesthesia team in charge. All patients received standard anesthetic and perioperative care. Choice of anesthetic agents, muscle relaxants, perioperative analgesia and prophylactic antibiotics was left to the discretion of the anesthesiologist in charge and only N_2_O administration and concentration was defined according to group random allocation. Additional neuraxial and other regional anesthetic techniques were accepted. Anesthesiologists were expected to maintain oxygenation, heart rate and blood pressure within the patient’s usual range at all times perioperatively and were advised to avoid intraoperative hypothermia (Temperature < 36 °C).Supplemental O_2_ could be administered at any time if required by impaired gas exchanges.

On the day of surgery, patients were assessed by a neuropsychologist in recovery for the presence of acute postoperative delirium according to the DSM-IV criteria [Agitation/restlessness; disorientation; speech confusion; attention deficit].

On day 7 following surgery, cognitive test administrators used the same computerized battery of tests (CANTAB) as in the preoperative period. To minimize learning effect and testing administrator bias, a parallel 1 version of PRM tests was used and the same administrator performed the testing process. On day 90 following surgery, the same computerized battery of tests (CANTAB) was used in a parallel 2 version of PRM test.

The CANTAB tests were administered either at the hospital or at home, if patients had been discharged. Quality of life was also measured using the EuroQol test (EQ-5D questionnaire http://www.euroqol.org/. When adverse events or unexpected outcomes were detected, further testing and clinical review were also organized.

We recorded patient demographics, risk factors, ASA scores, medication and all perioperative events and complications. To identify possible confounders for cognitive disorders, we measured anxiety and depression using the hospital anxiety and depression scale (HAD), the verbal pain score (VRS) and recorded all benzodiazepine use, cortisone, alcohol consumption opiates, antidiabetic drugs, non-tricyclic antidepressant drugs, antihypertensive drugs, natremia before each cognitive testing session. We also recorded preoperative blood glucose, hemoglobin level, blood pressure, heart rate and body temperature.

### Study outcomes

The primary outcome was postoperative neurocognitive recovery at 7 days and testing repeated at 90 days. It was defined as the within subject change between preoperative (baseline) and postoperative scores for each of the outcome measures selected within the following CANTAB tests: 1) PRM (Pattern recognition memory) test; 2) RTI (reaction time) test; 3) OTS (One Touch Stocking of Cambridge) test. Details of the tests are provided in “Supplementary 1: Appendix 1”.

The secondary outcomes were postoperative delirium, the number of unplanned intensive care unit (ICU) admissions, duration of hospital stay and quality of life using the EQ-5D questionnaire^25^. We also measured all adverse events occurring after surgery in both groups. Study data collected were stored locally in a locked database before being all securely transferred to the Geneva Study Centre. After all queries from the database manager were answered, individual center data were cleaned, aggregated and finally transferred to the statistical unit of the Center for Clinical Research.

### Sample size calculation

The sample size calculation was based on a cohort study assessing deterioration in cognitive performance (more specifically memory) following non-cardiac surgery^16^. To detect a 11.8% difference in cognitive impairment at 7 days with an increase from 15.7% to 27.5% between patients receiving or not N_2_O during anesthesia, we calculated a need for 190 patients per group, with 80% power and a significance level of 5%. To account for two planned interim analysis and the loss of follow-up (assumed to be limited) we targeted an overall recruitment of 420 patients. A constant likelihood group sequential method with formal futility boundaries was used with a two-sided Pocock stopping rule. There was no contingency for early termination for efficacy. An acceptance region plot (or a futility region plot) was generated using Spotfire SeqTrial for S+, TIBCO Spotfire S+ Version 8.2.0 for Windows, TIBCO Software Inc, Palo Alto, CA, USA https://edelivery.tibco.com/storefront/eval/tibco-spotfire-s-/prod10222.html. The two-sided futility boundary (for the differences in proportions between the N2O and the Air/Oxygen group) at planned interim analysis T1 (N = 140) was from -0.0244 to + 0.0244 and at analysis T2 (N = 280) from -0.0696 to 0.0696 (Supplementary 2: Appendix 2).

### Statistical analysis

The trial data and safety monitoring committee monitored compliance and the analysis of study results. Because repeated cognitive testing following anesthetic care can generate significant anxiety in patients, two interim analyses assessing episodic memory (PRM test) were scheduled. One after enrolment of 140 and another after 280 patients. The interim analysis was adjusted according to the Pocock Type I error function. The futility boundaries were ± 0.0244 at the first and ± 0.0696 at the second analysis for the between trial groups difference at seven days in the primary outcome (proportion of successful results for immediate and delayed PRM tests)^26^.

Categorical variables were summarized as frequencies and percentages and continuous variables as means with standard deviation (SD). For primary and secondary outcomes, changes from baseline were compared between groups using the χ^2^ test or Student t test or Mann–Whitney test as appropriate. A modification of the intervention effect between days 7 and 90 was investigated by using mixed effect linear regression models with a random intercept considering only timing (day 90 or day 7) and study group (N2O vs Air/O_2_) as independent variables with fixed effects and an interaction term. For co-variates analysis we used multivariate linear regressions and an interaction between timing and study group was tested in a linear model with mixed effects^27^. Group allocation, duration of surgery and average concentration of sevoflurane used were introduced into the model.

Possible collinearity was tested and could be formally excluded. The final results are expressed as adjusted 95% CI and P values. A P-value of < 0.05 was considered statistically significant. We used the Statistical Package for Social Sciences-SPSS (Version 22, SPSS, Inc., Chicago-Illinois/US) and R (release 2.13.1; R Foundation for Statistical Computing, Vienna, Austria).

## Results

### Study course

At interim analysis 1, investigators submitted data to the Data Safety and Monitoring Committee. For the primary outcome used for sample size calculation (episodic memory[PRM test] :proportion of correct answers immediate and delayed) the between group difference observed at 7 days was < 0.0244 and within the stopping boundaries for futility at T1. Study recruitment proved also to be particularly difficult and resources falling short. Based on both arguments, the committee decided to stop the trial.

**Figure 1:**
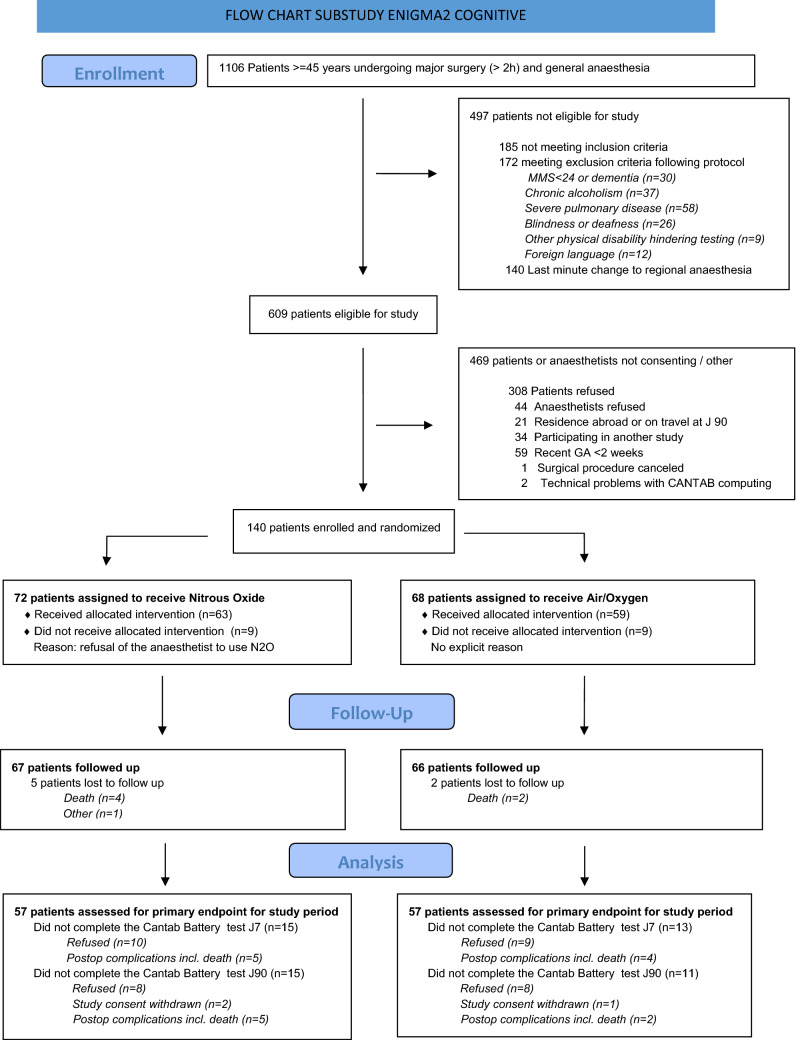
Study flow chart.

During the study period, 609 patients were found eligible for the study of which 140 consented to be randomized; 68 patients were in the Air/O_2_ study arm and 72 in the N_2_O/O_2_ group. By day 90, seven patients were lost to follow up (5 in the group Nitrous Oxide and 2 on group Air/Oxygen). The whole cognitive testing process (preoperative, day 7, day 90) could be achieved in 114 patients. In the remaining group of 28 patients, only preoperative, day 7 or day 90 testing could be performed. The study flow chart is provided in Fig. 1.

All randomized patients were included in the statistical analysis and analyzed as complete cases. Both intention-to-treat and as-treated analyses for outcome measures of the CANTAB test were performed. Results are provided in “Supplementary 3: Appendix 3”.

### Study participants’ characteristics

Baseline characteristics such as age, education level, MMSE and HAD tests scores were similar between groups (Table 1). Study patient mean age was 70.5 years in the Air/O_2_ group and 69.4 years in the N_2_O group. About two-thirds of patients were men. Alcohol consumption, benzodiazepine or antidepressant drugs use was similar in both groups. The mean inspired oxygen concentration did not differ between groups (P value = 0.18).

**Table 1:** Demographic data comparing patient in the N_2_O versus N_2_O-free anesthesia group.

	Air/oxygen (n = 68)	N_2_O (n = 72)
Age, years mean(SD)	70.5 (7.8)	69.4 (7.4)
Men, n (%)	46 (67.6)	48 (66.7)
Bodyweight, kg mean(SD)	69.2 (16.0)	70.6 (16.9)
**Ethnicity,** n (%)		
Asian	40 (60.6)	40 (55.6)
European	26 (39.4)	32 (44.4)
**ASA physical status score,** n (%)		
II	42 (61.8)	47 (65.3)
III	26 (38.2)	25 (34.7)
**MMSE score,** mean(SD)	27.1 (1.7)	27.1 (1.6)
**Education level,** n (%)		
Elementary School or less	19 (27.9)	23 (31.9)
Lower secondary School or equivalent (i.e. professional training)	32 (47.1)	28 (38.9)
Upper secondary School or post-secondary non tertiary education	10 (14.7)	11 (15.3)
Tertiary education level (Bachelor, Master, Doctorate) or other equivalent	7 (10.3)	10 (13.9)
**Pre-existing medical conditions,** n (%)		
Hypertension	56 (82.4)	64 (88.9)
Coronary artery disease	11 (16.2)	16 (22.2)
Heart failure	12 (17.6)	13 (18.1)
Previous myocardial infarction	3 (4.4)	9 (12.5)
Previous CABG or PCI	5 (7.4)	7 (9.7)
Peripheral vascular disease	9 (13.2)	19 (26.4)
Previous stroke or TIA	10 (14.7)	10 (13.9)
Diabetes	32 (47.1)	31 (43.1)
Current infection or fever	2 (2.9)	1 (1.4)
**Dietary factors,** n (%)		
Vegan or vegetarian	0 (0.0)	0 (0.0)
Regular folate use	3 (4.4)	4 (5.6)
Vitamin B12 supplementation	0 (0.0)	2 (2.8)
**Preoperative medications,** n (%)		
ACE inhibitors ARBs	34 (50)	41 (56.9)
Amiodarone	1 (1.5)	0 (0)
Beta-blockers	21 (30.9)	27 (37.5)
Ca2 blockers	35 (51.5)	35 (48.6)
**Preoperative laboratory tests**		
Intra-op blood glucose, mmol/l^−1^	7.0 (2.6)	6.8 (2.4)
Hemoglobin, g/l^−1^	12.8 (2.2)	12.7 (1.8)

Data are presented as mean (± SD) for age, Bodyweight, MMSE score, Blood glucose, Haemoglogin level. All other variables are N (%).

NA, not assessable.

There was no between group difference in procedure related data except a longer duration of surgery (35 min on average) and a higher concentration of sevoflurane consumption (expired concentration) in the Air/O_2_ group [1.2% vs. 0.7% ] (Table 2).

**Table 2:** Procedure and postoperative related data comparing patient in the N_2_O versus N_2_O-free anesthesia group.

	Air/oxygen (n = 68)	N_2_O (n = 72)
Lowest BIS	35.1 (9.0)	34.8 (8.8)
Highest BIS	71.3 (11.1)	69.9 (12.5)
SpO_2_	96.5 (3.1)	96.7 (2.4)
Lowest SBP	87.2 (13.9)	87.2 (13.4)
Duration of surgery, min	249 (123)	213 (79)*
**Anaesthesic drugs, n(%)**		
Midazolam	6 (8.8)	10 (13.9)
Fentanyl	40 (58.8)	38 (52.8)
Morphine	16 (23.5)	15 (20.8)
Ketamine	7 (10.3)	9 (12.5)
Other opioid	33 (48.5)	35 (48.6)
Beta blockers	5 (7.4)	10 (13.9)
Alpha blockers	3 (4.4)	2 (2.8)
Dexamethasone	3 (4.4)	2 (2.8)
5HT3Antagonist	23 (33.8)	29 (40.3)
Droperidol	2 (2.9)	1 (1.4)
Intra-Op opioid^a^	68 (100)	72 (100)
FIO_2_	37.5 (6.5)	35.8 (8.3)
FE Sevoflurane, %	1.2 (0.6)	0.7 (0.5)*
FE Isoflurane, %	0.02 (0.1)	0.03 (0.1)
FE Desfluran, %	0.15 (0.8)	0 (0)
**Postoperative data**		
Recovery room blood glucose, mmol/l	7.9 (1.8)	8 (2.0)
VRS Pain Score	1.4 (2.4)	1.2 (2.1)
Alcohol consumption, n%	17 (25.0)	25 (34.7)
Benzodiazepine, n%	2 (2.9)	7 (9.7)
Cortisone, n%	4 (5.9)	1 (1.4)
Insulin, n%	9 (13.2)	4 (5.6)
Oral hypoglycemic medications, n%	24 (35.3)	29 (40.3)
Nontricyclic antidepressants, n%	6 (8.8%)	6 (8.3%)
HAD Anxiety Score	4.1 (3.7)	4.2 (3.8)
HAD Depression Score	3.3 (3.6)	3.20 (3.2)

All data are presented as mean (± SD) except when specified N, (%).

P < 0.05 following *t test for comparisons of means and Chi-2 test (or Fisher exact test) for comparisons of proportion.

^a^Yes if intra-op fentanyl, morphine or other opioids.

### Between group difference for primary outcomes

For outcome measures of the PRM test (episodic memory), the mean (95% CI) between group difference for the change in the proportion of correct answers (immediate recall) was -1.5% (-7.1 to 4.0), P = 0.583. At Day 90, it was -0.9% (-6.8 to 4.9), P = 0.744. For delayed recall at Day 7, the mean (95% CI) difference was 2.9% (-4.1 to 10.0), P = 0.406. At Day 90, it was 4.4% (-2.1 to 11.0), P = 0.182 (Table 3). These fell in the futility margins of the Pocock boundaries (Supplementary 2: Appendix 2).

**Table 3:** Group differences for outcome measures of the pattern recognition memory test (PRM).

	Air/oxygen^a^ (n = 68)	N_2_O^a^ (n = 72)	Mean difference (95%CI)	P value**
**PRM pci, mean %**				
Preop (baseline)	81.2 (17.0)	83.2 (15.6)	− 2.0 (− 7.4 to 3.5)	
Change from baseline at day 7	− 1.4 (15.3)	0.1 (14.5)	− 1.5 (− 7.1 to 4.0)	0.583
Change from baseline at day 90	− 2.5 (16.5)	− 1.6 (14.6)	− 0.9 (− 6.8 to 4.9)	0.744
**PRM tci, msec**				
Preop (baseline)	2977 (1204)	2913 (1055)	64.0 (− 319.0 to 446.0)	
Change from baseline at day 7	− 302.0 (1430.1)	− 200.3 (1192.4)	− 101.7 (− 577.2 to 373.8)	0.672
Change from baseline at day 90	− 350.1 (997.4)	− 86.6 (2533.6)	− 263.5 (− 1014.8 to 487.8)	0.486
**PRM pcd, mean %**				
Preop (baseline)	76.0 (16.3)	79.4 (14.8)	− 3.4 (− 8.6 to 1.8)	
Change from baseline at day 7	2.8 (19.9)	− 0.1 (17.4)	2.9 (− 4.1 to 10.0)	0.406
Change from baseline at day 90	0 (15.8)	− 4.4 (19.1)	4.4 (− 2.1 to 11.0)	0.182
**PRM tcd, msec**				
Preop (baseline)	3273 (1240)	3234 (1321)	39 (− 392 to 470)	
Change from baseline at day 7	− 350 (978)	− 460 (1032)	110 (− 439 to 660)	0.688
Change from baseline at day 90	− 276 (955)	− 579 (1226)	303 (− 289 to 894)	0.310

PCI, proportion of correct answers immediate or the mean proportion of correct answers in the immediate test; TCI, time to correct answers immediate or the mean time to provide correct answers in the immediate test; PCD, proportion of correct answers delayed or the mean proportion of correct answers in the delayed test; TCD, time to correct answers delayed or the mean time to provide correct answer in the delayed test.

**Change from baseline was compared between trial groups with t tests. In addition, a modification of the effect from day 7 to day 90 was tested with an interaction term in a linear regression model with mixed effects and was not found statistically significant (p-values from 0.16 to 0.51).

^a^All data are presented as mean (± SD).

For outcome measures of the RTI test (decision making and processing speed) there was no between group difference at day 7 or day 90. At 7 days, the mean (± SD) reaction time change from baseline (single stimulus mode) was 19.4 (85.5) msec in the Air/O2 group and 43.3 (164.7) msec in the N2O group. The mean (95% CI) between group difference was -23.9 (-75.2 to 27.4), P = 0.356 (Table 4).

**Table 4:** Group differences for outcome measures of the reaction time test (RTI).

	Air/oxygen^a^ (n = 68)	N2O^a^ (n = 72)	Mean difference (95%CI)	P-value**
**RTI srt, msec**				
Preop (baseline)	377.2 (168.2)	367.0 (106.3)	10.2 (− 38.6 to 59.2)	
Change from baseline at day 7	19.4 (85.5)	43.3 (164.7)	− 23.9 (− 75.2 to 27.4)	0.356
Change from baseline at day 90	− 24.8 (202.6)	− 5.0 (148.2)	− 19.8 (− 89.5 to 49.7)	0.572
**RTI smt**, **msec**				
Preop (baseline)	573.9 (163.5)	571.8 (231.4)	2.1 (− 67.6 to 71.7))	
Change from baseline at day 7	64.5 (200.4)	17.4 (211.7)	47.1 (− 34.3 to 128.5)	0.253
Change from baseline at day 90	− 30.6 (176.2)	5.3 (300.8)	− 25.3 (− 132.3 to 60.5)	0.460
**RTI sascore, mean**				
Preop (baseline)	10.8 (3.3)	11.2 (3.5)	− 0.4 (− 1.5 to 0.7)	
Change from baseline at day 7	0.1 (1.63)	0.19 (1.3)	− 0.09 (− 0.6 to 0.5)	0.950
Change from baseline at day 90	− 0.04 (1.61)	0.13 (1.21)	− 0.17 (− 0.7 to 0.3)	0.537
**RTI 5rt, msec**				
Preop (baseline)	409.3 (122.1)	384.8 (73.8)	24.5 (− 10.8 to 59.7)	
Change from baseline at day 7	23.8 (140.1)	34.9 (155.9)	− 11.1 (− 69.6 to 47.3)	0.706
Change from baseline at day 90	− 22.8 (135.5)	− 6.7 (103.4)	− 16.1 (− 64.13 to 32.0)	0.507
**RTI 5mt, msec**				
Preop (baseline)	552.0 (180.7)	533.2 (242.3)	18.8 (− 56.0 to 93.6)	
Change from baseline at day 7	27.9 (145.4)	− 1.2 (252.2)	29.1 (− 52.3 to 110.5)	0.148
Change from baseline at day 90	− 50.6 (239.3)	1.4 (334.2)	− 49.2 (− 167.5 to 63.6)	0.569
**RTI 5sacore, mean**				
Preop (baseline)	10.4 (3.8)	10.8 (3.9)	− 0.4 (− 1.7 to 0.9)	
Change from baseline at day 7	0.19 (1.7)	0.09 (1.6)	0.10 (− 0.5 to 0.7)	0.778
Change from baseline at day 90	0.18 (1.6)	0.29 (1.5)	− 0.11 (− 0.7 to 0.5)	0.729

RTIsrt, simple reaction time (msec) or speed of press pad release following single stimulus; RTIsmt, simple movement time (msec) or time to press a single stimulus after press pad release, RTI sascore, simple accuracy score or total number of correct trials out of 15 for a single stimulus; RTI 5rt, five choice reaction time (msec) or speed of press pad release following five different stimulus; RTI 5mt, five choice movement time (msec) or time to press one out of five stimulus after press pad release, RTI 5sacore, five choice accuracy score or total number of correct trials out of 15 for a five choice stimulus.

**Change from baseline was compared between trial groups with t tests. In addition, a modification of the effect from day 7 to day 90 was tested with an interaction term in a linear regression model with mixed effects and was not found statistically significant (p-values from 0.16 to 0.95).

^a^All data are presented as mean (± SD).

For outcome measures of the OTS test (executive functions) we found a significant between group differences at 7 days (Table 5). The mean (± SD) change from baseline for the total number of problems successfully solved on first choice (out of 20) was -0.1 (2.6) in the Air/O_2_ group and 1.2 (2.1) in the N_2_O group. The mean (95% CI) between group difference was -1.3 (-2.7 to -0.1), P = 0.048 in favor of N_2_O. The mean (± SD) change from baseline for the number of box choices made to correctly solve the problem was 0.01 (0.1) in the Air/O_2_ group and -0.1 (0.1) in the N_2_O group. Mean (95% CI) between group difference was 0.11 (0.01 to 0.20), P = 0.029 in favor of N_2_O. At Day 90, the between group differences disappeared showing marginal differences with preoperative values, particularly for the RTI test, suggesting a full recovery of preoperative function at 3 months.

**Table 5:** Group differences for outcome measures of the one touch stocking test (OTS).

	Air/oxygen^a^ (n = 68)	N_2_O^a^ (n = 72)	Mean difference (95%CI)	P-value**
**OTS mn1c, mean**				
Preop (baseline)	15.4 (2.0)	13.8 (2.7)	1.6 (0.6 to 3.1)	
Change from baseline at day 7	− 0.1 (2.6)	1.2 (2.1)	− 1.3 (− 2.7 to − 0.1)	0.048
Change from baseline at day 90	0.5 (2.2)	0.9 (2.5)	− 0.4 (− 1.7 to 0.9)	0.547
**OTS m1c, mean**				
Preop (baseline)	1.3 (0.1)	1.4 (0.2)	− 0.1 (− 0.2 to 0.01)	
Change from baseline at day 7	0.01 (0.1)	− 0.10 (0.1)	0.11 (0.01 to 0.2)	0.029
Change from baseline at day 90	− 0.05 (0.1)	− 0.08 (0.1)	0.03 (− 0.07 to 0.1)	0.405
**OTS L1c, msec**				
Preop (baseline)	30,164 (18,195)	41,283 (33,489)	− 11,119 (− 28,438 to 461)	
Change from baseline at day 7	− 5409 (7801)	− 10,169 (14,437)	4760 (− 1523 to 11,042)	0.134
Change from baseline at day 90	− 10,829 (9650)	− 12,812 (17,721)	1983 (− 5853 to 9819)	0.612

OTS N1C, Mean Number first choice or the mean of the total number of problems out of 20 solved on first choice; OTS M1C, Mean first choice or the total mean number of unique box choices made on each of the 6 problems to find correct solution; OTS L1C, Latency first choice or the mean latency time between ball appearance and screen touch for unique box choices made on each of the 6 problems to find correct solution.

**Change from baseline was compared between trial groups with t tests. In addition, a modification of the effect from day 7 to day 90 was tested with an interaction term in a linear regression model with mixed effects and was not found statistically significant (p-values from 0.12 to 0.46).

^a^All data are presented as mean (± SD).

### Covariates analysis and secondary outcomes

To adjust for confounding factors we selected covariates that were imbalanced between treatment and control groups and likely to be significantly related to the outcome (cognitive function)^27^. Following multivariate adjustment for duration of surgery and the concentration of sevoflurane (higher in the Air/O_2_) group, we found a persisting difference for the OTS test (P = 0.042 and P = 0.026). Results are provided in Table 6. As-treated and intention to treat analyses did not meaningfully differ (Supplementary 2: Appendix 2).

**Table 6:** Adjusted Group differences for outcome measures of the OTS.

		OTS MN1C		OTS M1C		OTS L1C	
		Problem solved on 1st choice		Mean choice to correct		Mean latency to correct	
		Estimate (se)	p	Estimate (se)	p	Estimate (se)	p
**Change from baseline to day 7**							
Intercept		− 0.5 (1.1)	0.657	0.04 (0.08)	0.616	− 4656 (5754)	0.422
Group	Air/oxygen	0 (ref)		0 (ref)		0 (ref)	
	N2O	1.4 (0.7)	0.042*	− 0.1 (0.05)	0.026*	− 4894 (3570)	0.176*
**Change from baseline to day 90**							
Intercept		1.6 (1.1)	0.176	− 0.09 (0.09)	0.321	− 14,658 (7601)	0.059
Group	Air/oxygen	0 (ref)		0 (ref)		0 (ref)	
	N2O	− 0.03 (0.7)	0.962*	− 0.01 (0.05)	0.845*	− 39 (4576)	0.993*

*Adjusted for sevoflurane (concentration per unit) and duration of surgery (per hour) in multiple regression linear models. In addition, a modification of the effect from day 7 to day 90 was tested with an interaction term in a linear regression model with mixed effects and was not found statistically significant (p-values from 0.12 to 0.45).

In the analysis of secondary outcomes, we found that the number of unplanned ICU admissions was higher in the Air/O_2_ group than in the N_2_O group, 12 (17.6%) vs 5 (6.9%). This just not reach statistical significance (P = 0.05).

The duration of hospital stay, the number of patients with acute postoperative delirium, utility and pain scores for EuroQoL 5D were similar between both groups. The number of adverse events did not differ between both groups either, P = 0.66. (Table 7).

**Table 7:** Group differences for secondary outcomes and adverse events.

	Air/oxygen N = 68	N_2_O n = 72	OR (95%CI)	p value
Unexpected ICU admissions, n(%)	12 (17.6)	5 (6.9)	0.3 (0.1–1.0)	0.05
Duration of hospital stay, days	7.5 (5.7)	6.9 (3.9)	NA	0.52 *
**Postoperative confusion (DSM V)**, **n(%)**				
At least 1 symptom	11 (16.2)	8 (11.1)	1.4 (0.6 to 3.6)	0.53
All symptoms	1 (1.5)	0 (0)	∞	0.48
**QoL(EQ-5D)**				
Utility score	58.4 (10.3)	56.7 (10.4)	NA	0.35
EQ-VAS (pain)	72.4 (19.7)	73.4 (16.8)	NA	0.76
**Any adverse event , n(%)**				
Mild	4 (5.8)	9 (12.5)	1.0	0.66
Moderate	5 (7.3)	5 (6.9)	2.2 [0.4—10.8]	
Severe	7 (10.2)	7 (9.7)	1.0 [0.1—5.0]	
**Details of categories of adverse events**				
Neurological	0	0		
Respiratory	1	6		
Gastrointestinal	1	1		
Cardiovascular	4	2		
Renal failure/dysfunction	1	0		
Bleeding complications	1	2		
Skin	1	0		
Musculoskeletal/arthritis	0	0		
Local infection-sepsis	2	0		
Unplanned reoperation	4	0		
Anaemia	5	4		
Metabolic or electrolyte disturbances	0	1		
Hospital readmission	2	1		

*Mann-Whitney U test for comparisons of means (non parametric) and Chi-2 test (or Fisher exact test) for comparisons of proportions.

## Discussion

### Main results

The study purpose was to compare postoperative neurocognitive recovery in patients receiving N_2_O or N_2_O-free anesthesia. For all outcome measures of the CANTAB tests used for episodic memory and decision making/processing speed assessment, there were no between group differences at 7 and 90 days following surgery, suggesting harmlessness of N2O. Surprisingly, outcome measures of executive function tests significantly differed at 7 days. Patients receiving N_2_O had improved postoperative neurocognitive recovery compared with patients receiving Air/Oxygen. This finding cannot be explained by preexisting differences in the level of preoperative anxiety and depression (HAD), age, cognitive reserve or education level^28,29^ since all were equal between groups. For secondary outcomes (duration of hospital stay, delirium, unplanned admissions to the ICU, utility and pain scores for EuroQoL 5D) no difference was found between the two groups.

### Possible explanations for associations identified or not between cognitive tests results and N_2_O administration

Our study findings significantly differ from those from animal studies which describe impaired memory and learning as well as neuro behavioral disturbances following anesthesia with N_2_O. A detrimental effect of nitrous oxide on brain function is advocated in these studies to explain these findings^13,30–32^. However, in these animal studies, subjects were submitted to long durations of exposure to N_2_O (between 4 to 8 h) and to the combined administration of several different anesthetic drugs and gases such as isoflurane. As a result the specific contribution of N_2_O to cognitive changes following anesthesia is difficult to demonstrate^33^. In studies on humans, only a limited number of studies (cases reports and small size studies) seem to support the evidence of the noxious effect of N_2_O on brain function^14,34,35^. These studies find modified psychomotor performance after surgery, particularly reduced reaction time and short term memory in patients having received N_2_O during their anesthesia. In one case report, severe lesions of the brain with nerve demyelination following the administration of N_2_O is even described^36^. However, large observational studies and randomized trials do not seem to confirm these findings, including in the long term (i.e. 3 months)^16,18–20,37^. Likewise in our trial, we did not find evidence of a detrimental effect of N_2_O on memory or reaction time, including at 90 days. In contrast, we found that patients receiving N_2_O compared with patients receiving Air/O_2_ had an improved recovery process of executive functions at seven days after surgery.

Some other authors in animal studies describe a neuroprotective effect of N_2_O^21^ and Leung JM et al^19^, found in a randomized trial involving 228 elderly patients a higher (but non-significant, P = 0.59) incidence of postoperative cognitive disorder in the Air/ O_2_ group compared with the N_2_O group (18.6% vs 14.8%). This finding similar to the one in our study requires further discussion. The association between an improved recovery process of executive functions following surgery and the use of N_2_O for anesthesia is quite unexpected.

Several hypotheses can be formulated to explain this finding. One is the sparing effect of N_2_O when used in combination with other anesthetic agents such as isoflurane or sevoflurane. It allows the use of lower concentrations of volatile anesthetics and consequently decreases the risk of detrimental cognitive side effects often observed when high doses of isoflurane or sevoflurane are used^38,39^. However, this cannot explain our study findings. Following multivariate adjustment for volatile anesthetic concentration and duration of surgery, a significant difference could still be observed for the OTS test. This suggests an independent effect of N_2_O on the recovery of executive functions after anesthesia. Another possible explanation is a regression to the mean phenomenon. Patients in the N_2_O group started with lower preoperative scores for the OTS test compared with patients receiving Air/O_2._ Their postoperative improvement could be simply explained by a natural variation following repeated testing. Another explanation could be the selective blockage of *N*-methyl-D-aspartate (NMDA) receptors and a possible neuroprotective effect of N_2_O. The NMDA channel allows the influx of Ca^2+^ into specific brain cells. This mechanism is considered as critical for synaptic plasticity and it can affect both circuit and brain function^40,41^. When extra synaptic NMDA receptors are over-activated by high glutamate secretion (i.e. stroke, brain ischemia) an excessive influx of Ca^2+^occurs, leading to excitotoxicity and progressive cellular death^42^. Blocking NMDA receptors could therefore protect ischemic neurons from cellular death and promote functional recovery^43,44^.

Since a high number of NMDA receptors are located in the prefrontal cortex involved in executive functions, their selective blockade by N_2_O may have protected this cerebral area from the effect of intraoperative stress or hypoxemia. Subgroup analyses of the IHAST Trial^45^ show that patients in the N_2_O group could be discharged home earlier and had improved recovery^46^. However further studies are needed to confirm this hypothesis. Another interesting finding of our study, although just not statistically significant, is the lower number of ICU admissions in patients receiving N_2_O. While this may be due to chance, it could also be the result of a lower number of postoperative complications in patients receiving N_2_O^47^. The mechanism to explain this finding is however unclear.

### Strengths and limitations

A limitation of our study is the premature discontinuation of patient recruitment at first interim analysis. The trial fell in the futility margins of the Pocock boundaries for outcome measures of the PRM test, with no difference identifiable in episodic memory change between the two groups compared. There was however a significant difference in cognitive function recovery, using the OTS test. Yet this outcome had not been used for sample size and interim analysis boundaries estimations. Thus this result may be due to chance following interim result analysis and a fully completed trial may possibly not confirm this finding. Furthermore, we found that patients in the N_2_O group not only recovered better but even significantly improved their OTS score following surgery. This may seem counterintuitive and lead to the conclusion of an incorrect finding. This cannot be excluded. However a meta-analysis of patients having Coronary By Pass Graft (CABG) surgery^48^ confirms that cognitive performance can improve following surgery. This may be due to the beneficial effect of surgery on overall inflammatory status (once diseased organs have been removed) or to a learning effect of the cognitive tests administered^29^. This is particularly true when using tests sensitive to changes in the speed of psychomotor function. The OTS test used in our study is very sensitive to such changes. It assesses a fine set of cognitive abilities such as planning, decision making and impulse control. These functions involve for a large portion brain cells that are located in the pre-frontal cortex and that can easily be altered by perioperative stress, inflammation or transitory hypoxemia^49,50^. Significant improvement may be observed, particularly in patients ﻿receiving N_2_O. A third limitation of the trial is the confounding effect of the learning phenomenon when the same tests are performed several times. Participants’ performance automatically improves by learning. Score differences in cognitive testing may therefore reflect a learning effect rather than a true difference in cognitive function. But since we used, whenever possible, parallel versions of the CANTAB cognitive tests, a learning phenomenon is quite unlikely to explain our study results. The fourth limitation is the “ceiling effect”. It is observed in tests based on relatively easy tasks to perform. Patients can easily achieve a high score during the initial phase of the testing (for example preoperatively in our study) and a slight change in further testing may consequently not be detected since initial scores are already quite high. This increases the risk of type 2 errors in studies. In our trial, preoperative results of the reaction time test (RTI) were relatively high with a simple accuracy score of respectively 10.8 (3.3) and 11.2 (3.5) in the Air/O_2_ and N_2_O groups. This is however quite unlikely to have had an impact on our study results since we used several different outcome measures to compare each group. Many of these outcome measures are unlikely to be affected by a “ceiling effect” since they include no predefined upper limits (i.e. processing speed, latency time). A ceiling effect cannot occur since no maximum score can be reached.

Another limitation is the relatively low level of education of participants (two thirds had lower secondary or elementary school level). This may be responsible of performance limitations at the lower ranges of the tests, making decline more difficult to detect, particularly for tests that are less sensitive such as the PRM test. To minimize this effect we chose to define cognitive change following administration of N2O as a within individuals and between groups difference of either 1 SD or at least 25% difference in 1 or 2 tests. We also compared group differences using t tests for statistical differences ^51^.

Despite these limitations we found that N_2_O had no impact on postoperative episodic memory and processing speed functions at 7 days and 3 months following surgery. Patients who received N_2_O appeared even to have improved recovery of executive function at seven days. Due to the limitations of this interim study analysis finding, further studies are however needed to confirm a possible neuroprotective effect of N_2_O administered during anesthesia.

In conclusion, while confirming the harmlessness of N_2_O on executive memory and processing speed function this study opens interesting clinical and research perspectives on a possible use of N2O for high risk surgery, for patients with brain trauma or those having prolonged sedation in ICU care units and requiring neuroprotection.

## Supplementary Information

Supplementary Information 1.

Supplementary Information 2.

Supplementary Information 3.
