# Supplementary material for: The international ENIGMA-II substudy on postoperative cognitive disorders (ISEP)
Source: Sci Rep. 2021 Jun 2;11:11631. doi: 10.1038/s41598-021-91014-8 (PMC8173006; doi:10.1038/s41598-021-91014-8)

**Appendix 1**

**The International ENIGMA-II Substudy on Postoperative Cognitive Disorders (ISEP)**

**Guy Haller^1,3^** *MD, MSc, PhD.,* **Matthew TV Chan^2^** *MBBS, PhD, FHKCA, FANZCA, FHKAM* **Christophe Combescure^4^** *Msc,PhD*, **Ursula Lopez^5^** *Msc,PhD* **Isabelle Pichon^1^** *BN* **Marc Licker^1^** *MD* **Roxane Fournier^1^** *MD*, **Paul Myles^6^** *MBBS, MPH, MD, DSc, FCAI, FANZCA, FRCA, FAHMS*

1. Department of Acute Care Medicine, Division of Anesthesiology, Geneva University Hospitals and Faculty of Medicine, University of Geneva, Geneva, Switzerland

2. Department of Anaesthesia and Intensive Care, The Chinese University of Hong Kong, Prince of Wales Hospital, Shatin, New Territories, Hong Kong Special Administrative Region, China

3. Department of Epidemiology and Preventive Medicine, Health Services Management and Research Unit, Monash University, Melbourne Victoria, Australia

4. Department of Health and Community Medicine, Division of Clinical Epidemiology, University Hospitals of Geneva and Faculty of Medicine, University of Geneva, Geneva, Switzerland

5. Department of Medicine, Unit of Neuropsychology and Logopedics, Cantonal Hospital of Fribourg, Fribourg, Switzerland

6. Department of Anesthesiology and Perioperative Medicine, Alfred Hospital and Monash University, Melbourne Victoria, Australia

**DESCRIPTION OF THE TESTS SELECTED FROM THE CANTAB^®^**

**Test 1:** **Motor Screening (MOT)**


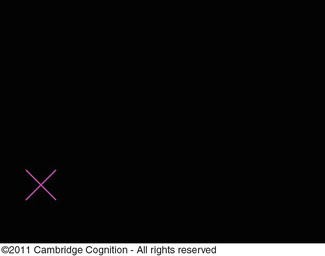


CANTAB® [Cognitive assessment software]. Cambridge Cognition (2021).

All rights reserved. www.cantab.com

This test is common to all of the CANTAB batteries. It is performed at the beginning of a test session. The purpose of the test is to familiarize patients with the computer and touch screen technology. It can screen for difficulties with fine motricity, vision and comprehension of simple instructions.

A series of crosses successively appear in different locations of the screen. The patient must point to each cross as soon as it appears, using his own forefinger.

Outcome measures for this test are:

a) Mean latency time between apparition of the cross and forefinger pressure

b) Mean number of errors defined as the mean distance between the centre of the cross and the location of patients screen touch.

**Test 2:** **Paired associated learning (PAL)**


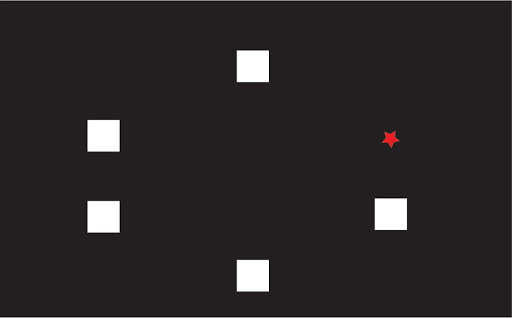


CANTAB® [Cognitive assessment software]. Cambridge Cognition (2021).

All rights reserved. www.cantab.com

Paired Associates Learning PAL are testing visual memory and new learning. This test is sensitive primarily to changes in the temporal and frontal lobes. In this test, initially six boxes are exhibited on the screen, and they are opened in a randomized order to reveal the contents. One or more of the boxes will contain a pattern. Each pattern is then displayed in the middle of the screen, one at a time, and the participant must identify the box containing the particular pattern. When the participant gets all the locations correct, he or she proceeds to the next stage, which includes eight boxes; otherwise, the test terminates. The test has an increasing level of difficulty ranging from two to eight patterns to be remembered [40]. The following are the outcome measures used in the PAL test: PAL total errors adjusted: The total number of errors made in all stages, along with an adjustment for each stage not attempted owing to previous failure PAL total errors (six shapes, adjusted): The total number of errors in the eight-stage pattern, with an adjustment made for subjects who have not reached this stage

**Test 3: Reaction Time (RTI) test**


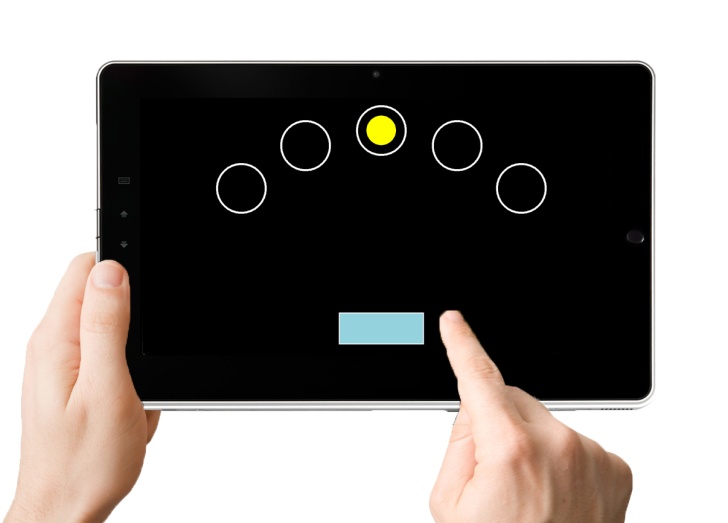


CANTAB® [Cognitive assessment software]. Cambridge Cognition (2021).

All rights reserved. www.cantab.com

This tests is designed to measure subjects’ processing speed. It assesses the speed of response to a visual target where the stimulus is either in a single (simple reaction time) or 5-choice mode (choice reaction time). The subject must hold a button at the bottom of the screen and release it as soon as possible to select a circle in which a dot has appeared. This test is a measure of general cerebral performance and is also sensitive to dysfunction in the parietal or frontal lobe areas of the brain.

Outcome measures for this test are

a) The speed of press pad release following single stimulus

b) The time to press a single stimulus after press pad release

c) The total number of correct trials out of 15 for a single stimulus

d) The speed of press pad release following five different stimulus

e) The time to press one out of five stimulus after press pad release

f) The total number of correct trials out of 15 for a five choice stimulus;

**Test 4: Pattern Recognition Memory test (PRM)**


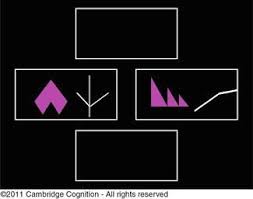


CANTAB® [Cognitive assessment software]. Cambridge Cognition (2021).

All rights reserved. www.cantab.com

This tests is designed to measure subjects’ episodic memory. It assesses the subjects’ ability to recognise visual patterns that have already been presented within minutes (immediate recall) or after a longer delay (delayed recall). Patterns are designed so that they cannot easily be given verbal names. This test is more sensitive to dysfunction in medial temporal areas but relatively insensitive to dysfunction in the frontal lobe.

Outcome measures for this test are

a) The mean proportion of correct answers (immediate)

b) The mean time to provide correct answers (immediate)

c) The mean proportion of correct answers (delayed)

d) The mean time to provide correct answer (delayed)

**Test 5:** **One touch stockings test (OTS)**


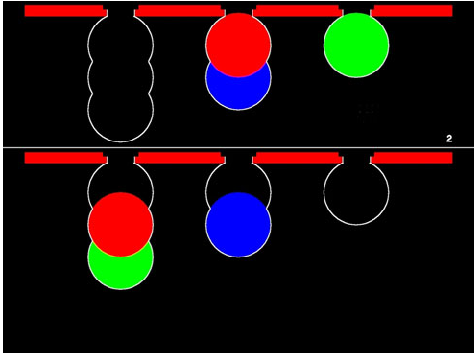


CANTAB® [Cognitive assessment software]. Cambridge Cognition (2021).

All rights reserved. www.cantab.com

This tests is designed to measure subjects’ executive function and more specifically spatial planning and working memory. It assesses subjects’ ability to reproduce a pattern made of stacks of coloured balls. There are 3 different colours and balls can be moved one at a time by touching the required ball and then touching the position to which it should be moved to. The number of required moves to complete the task increases in complexity from one move to five moves. In the next step of the exercise, the subject is shown more problems (up to a maximum of 20) and he must work out in advance how many moves are required. This test is sensitive to pre-frontal lobe damage
Outcome measures for this test are

a) The mean of the total number of problems out of 20 solved on first choice (higher is better)

b) The total mean number of box choices made to correctly solve the problem (the lower the number, the better)

c) The mean latency time between ball appearance and screen touch to find correct box to solve the problem

For outcomes b) and c), measures may be calculated for all problems, or for problems with a specified number of moves (1-move to 5 moves)

**Cerebral areas and cognitive functions dimensions assessed**

While each cognitive domain assessed by the different CANTAB battery tests refer to several areas of the brain that are activated during test performance some preferential locations can be identified and are shown below (Reproduced with permission of CANTAB® [Cognitive assessment software]. Cambridge Cognition (2021) All rights reserved. www.cantab.com)


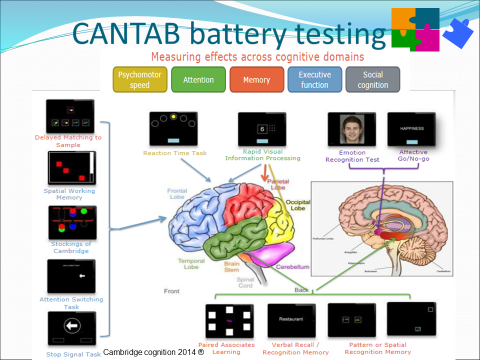

Supplement: Supplementary file 1 — Supplementary Information 1. [file 41598_2021_91014_MOESM1_ESM.docx]
