# Supplementary material for: The international ENIGMA-II substudy on postoperative cognitive disorders (ISEP)
Source: Sci Rep. 2021 Jun 2;11:11631. doi: 10.1038/s41598-021-91014-8 (PMC8173006; doi:10.1038/s41598-021-91014-8)
Supplement: Supplementary file 2 — Supplementary Information 2. [file 41598_2021_91014_MOESM2_ESM.docx]

**Appendix 2**

**The International ENIGMA-II Substudy on Postoperative Cognitive Disorders (ISEP)**

**Guy Haller^1,3^** *MD, MSc, PhD.,* **Matthew TV Chan^2^** *MBBS, PhD, FHKCA, FANZCA, FHKAM* **Christophe Combescure^4^** *Msc,PhD*, **Ursula Lopez^5^** *Msc,PhD* **Isabelle Pichon^1^** *BN* **Marc Licker^1^** *MD* **Roxane Fournier^1^** *MD*, **Paul Myles^6^** *MBBS, MPH, MD, DSc, FCAI, FANZCA, FRCA, FAHMS*

1. Department of Acute Care Medicine, Division of Anesthesiology, Geneva University Hospitals and Faculty of Medicine, University of Geneva, Geneva, Switzerland

2. Department of Anaesthesia and Intensive Care, The Chinese University of Hong Kong, Prince of Wales Hospital, Shatin, New Territories, Hong Kong Special Administrative Region, China

3. Department of Epidemiology and Preventive Medicine, Health Services Management and Research Unit, Monash University, Melbourne Victoria, Australia

4. Department of Health and Community Medicine, Division of Clinical Epidemiology, University Hospitals of Geneva and Faculty of Medicine, University of Geneva, Geneva, Switzerland

5. Department of Medicine, Unit of Neuropsychology and Logopedics, Cantonal Hospital of Fribourg, Fribourg, Switzerland

6. Department of Anesthesiology and Perioperative Medicine, Alfred Hospital and Monash University, Melbourne Victoria, Australia

**Results of boundaries calculation for interim analyis according to the Pocock model**

STOPPING BOUNDARIES: Sample Mean scale

                          a       b      c      d

**Time 1 (N= 140) -0.1552 -0.0244 0.0244 0.1552**

    Time 2 (N= 280) -0.1097 -0.0696 0.0696 0.1097

    Time 3 (N= 420) -0.0896 -0.0896 0.0896 0.0896

**Interim Analyses**


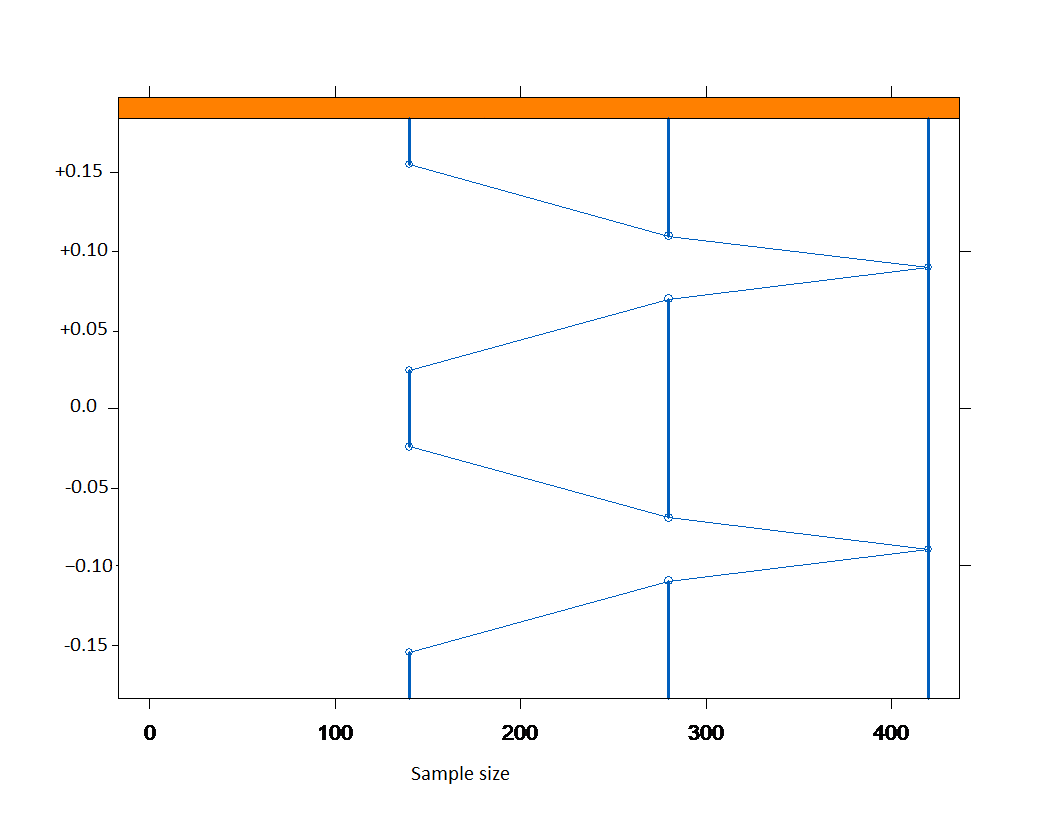


**0.0244**

**- 0.1552**

**- 0.0244**

**0.1552**

Trial continuation

Trial continuation

**Stopping for superiority**

**Stopping for futility**

**Stopping for inferiority**
