# Supplementary material for: The international ENIGMA-II substudy on postoperative cognitive disorders (ISEP)
Source: Sci Rep. 2021 Jun 2;11:11631. doi: 10.1038/s41598-021-91014-8 (PMC8173006; doi:10.1038/s41598-021-91014-8)
Supplement: Supplementary file 3 — Supplementary Information 3. [file 41598_2021_91014_MOESM3_ESM.docx]

**Appendix 3**

**The International ENIGMA-II Substudy on Postoperative Cognitive Disorders (ISEP)**

**Guy Haller^1,3^** *MD, MSc, PhD.,* **Matthew TV Chan^2^** *MBBS, PhD, FHKCA, FANZCA, FHKAM* **Christophe Combescure^4^** *Msc,PhD*, **Ursula Lopez^5^** *Msc,PhD* **Isabelle Pichon^1^** *BN* **Marc Licker^1^** *MD* **Roxane Fournier^1^** *MD*, **Paul Myles^6^** *MBBS, MPH, MD, DSc, FCAI, FANZCA, FRCA, FAHMS*

1. Department of Acute Care Medicine, Division of Anesthesiology, Geneva University Hospitals and Faculty of Medicine, University of Geneva, Geneva, Switzerland

2. Department of Anaesthesia and Intensive Care, The Chinese University of Hong Kong, Prince of Wales Hospital, Shatin, New Territories, Hong Kong Special Administrative Region, China

3. Department of Epidemiology and Preventive Medicine, Health Services Management and Research Unit, Monash University, Melbourne Victoria, Australia

4. Department of Health and Community Medicine, Division of Clinical Epidemiology, University Hospitals of Geneva and Faculty of Medicine, University of Geneva, Geneva, Switzerland

5. Department of Medicine, Unit of Neuropsychology and Logopedics, Cantonal Hospital of Fribourg, Fribourg, Switzerland

6. Department of Anesthesiology and Perioperative Medicine, Alfred Hospital and Monash University, Melbourne Victoria, Australia

**Table 1s: Group differences for outcome measures of the pattern recognition memory test (PRM)**

|  | **AIR/OXYGEN***  **(n=68)** | **N_2_O***  **(n=72)** | **Mean Difference (95%CI)** | **P-value**** |
| --- | --- | --- | --- | --- |
| **PRM pci, mean %**  Preop (Baseline)  Change from baseline at Day 7  Change from baseline at Day 90 | 81.7 (17.57)  -1.2 (15.5)  -2.5 (15.15) | 82.7 (15.09)  0 (14.35)  -1.6 (16.08) | -0.9 (-6.49 to 4.53)  -1.2 (-6.81 to 4.39)  -0.8 (-6.74 to 4.96) | 0.668  0.762 |
| **PRM tci, msec.**  Preop (Baseline)  Change from baseline at Day 7  Change from baseline at Day 90 | 3042.1 (1432.6)  -302.0 (1430.1)  -350.1 (997.4) | 2853.8 (739.8)  -200.3 (1192.4)  -86.6 (2533.6) | 188.3 (-202.2 to 578.8)  -101.7 (-533.9 to 416.6)  -263.5 (-1014.2 to 530.9) | 0.807  0.518 |
| **PRM pcd, mean %**  Preop (Baseline)  Change from baseline at Day 7  Change from baseline at Day 90 | 77.4 (16.78)  2.88 (20.11)  0.15 (16.17) | 78.1 (14.56)  -0.3 (17.11)  --4.7 (18.82) | -0.7 (-6.04 to 4.59)  3.1 (-3.86 to 10.21)  4.8 (-1.76 to 11.45) | 0.372  0.149 |
| **PRM tcd, msec.**  Preop (Baseline)  Change from baseline at Day 7  Change from baseline at Day 90 | 3337 (1423)  -351 (967)  -266 (937) | 3174 (1135)  -472 (1048)  -626 (1262) | 163 (-273 to 599)  121 (-430 to 674)  360 (-249 to 969) | 0.659  0.240 |

*

* All data are presented as mean (± SD) ; Abbreviations of test names are : PCI: proportion of correct answers immediate or the mean proportion of correct answers in the immediate test TCI: time to correct answers immediate or the mean time to provide correct answers in the immediate test PCD: proportion of correct answers delayed or the mean proportion of correct answers in the delayed test TCD: time to correct answers delayed or the mean time to provide correct answer in the delayed test

**: Change from baseline was compared between trial groups with t tests. In addition, a modification of the effect from day 7 to day 90 was tested with an interaction term in a linear regression model with mixed effects and was not found statistically significant.

**Table 2s: Group differences for outcome measures of the reaction time test (RTI)**

|  | **AIR/OXYGEN***  **(n=68)** | **N2O***  **(n=72)** | **Mean Difference (95%CI)** | **P-value**** |
| --- | --- | --- | --- | --- |
| **RTI srt, msec.**  Preop (Baseline)  Change from baseline at Day 7  Change from baseline at Day 90 | 383 (171)  36 (128)  -32 (208) | 361 (99)  28 (139)  3 (138) | 22.0 (-26.6 to 70.2)  8.0 (-44.5 to 60.9)  -35.0 (-103.3 to 34.5) | 0.758  0.323 |
| **RTI smt**, **msec.**  Preop (Baseline)  Change from baseline at Day 7  Change from baseline at Day 90 | 588 (210)  53 (188)  -45 (230) | 557 (189)  27 (225)  20 (260) | 31.0 (-37.5 to 100.8)  26.0 (-55 to 108.5)  -65.0 (-161.2 to 31.0) | 0.517  0.182 |
| **RTI sascore, mean**  Preop (Baseline)  Change from baseline at Day 7  Change from baseline at Day 90 | 11.2 (3.31)  0.24 (1.53)  -0.08 (1.52) | 10.9 (3.59)  0.12 (1.4)  0.18 (1.31) | 0.3 (-0.9 to 1.47)  0.12 (-0.4 to 0.7)  -0.26 (-0.8 to 0.3) | 0.669  0.366 |
| **RTI 5rt, msec.**  Preop (Baseline)  Change from baseline at Day 7  Change from baseline at Day 90 | 419.5 (130.2)  28.7 (193.1)  -40.9 (143.5) | 374.23 (49.9)  30.74 (88.9)  10.8 (85.3) | 48.3 (10.8 to 79.7)  -2.0 (-62.4 to 58.3)  -51.7 (-98.9 to -4.6) | 0.946  0.031 |
| **RTI 5mt, msec.**  Preop (Baseline)  Change from baseline at Day 7  Change from baseline at Day 90 | 383 (171)  36 (128)  -32 (208) | 361 (99)  28 (139)  3 (138) | 22.0 (-26.6 to 70.2)  8.0 (-44.5 to 60.9)  -35.0 (-103.3 to 34.) | 0.758  0.323 |
| **RTI 5sacore, mean**  Preop (Baseline)  Change from baseline at Day 7  Change from baseline at Day | 588 (210)  53 (188)  -45 (230) | 557 (189)  27 (225)  20 (260) | 31.0 (-37.5 to 100.8)  26.0 (-55 to 108.5)  -65.0 (-161.2 to 31.0) | 0.517  0.182 |

* All data are presented as mean (± SD) ; Abbreviations of test names are : RTIsrt – simple reaction time (msec) or speed of press pad release following single stimulus; RTIsmt – simple movement time (msec) or time to press a single stimulus after press pad release, RTI sascore – simple accuracy score or total number of correct trials out of 15 for a single stimulus; RTI 5rt – five choice reaction time (msec) or speed of press pad release following five different stimulus; RTI 5mt – five choice movement time (msec) or time to press one out of five stimulus after press pad release, RTI 5sacore – five choice accuracy score or total number of correct trials out of 15 for a five choice stimulus

**: Change from baseline was compared between trial groups with t tests. In addition, a modification of the effect from day 7 to day 90 was tested with an interaction term in a linear regression model with mixed effects and was not found statistically significant.

**Table 3s Group differences for outcome measures of the one touch stocking test (OTS)**

|  | **AIR/OXYGEN***  **(n=68)** | **N_2_O***  **(n=72)** | **Mean Difference (95%CI)** | **P-value**** |
| --- | --- | --- | --- | --- |
| **OTS mn1c, mean**  Preop (Baseline)  Change from baseline at Day 7  Change from baseline at Day 90 | 15.4 (2.1)  0.1 (2.5)  0.04 (2.0 | 13.6 (2.7)  1.0 (2.1)  1.4 (2.4 | 1.8 (0.6 to 3.1)  -0.9 (-2.2 to 0.5)  -1.4 (-2.7 to -0.1) | 0.198  0.031 |
| **OTS m1c,** **mean**  Preop (Baseline)  Change from baseline at Day 7  Change from baseline at Day 90 | 1.3 (0.1)  -0.03 (0.1)  -0.03 (0.1) | 1.4 (0.2)  -0.07 (0.1)  -0.11 (0.17) | -0.1 (-0.2 to 0.01)  0.04 (-0.0 to 0.1)  0.08 (-0.0 to 0.1) | 0.465  0.091 |
| **OTS L1c, msec**  Preop (Baseline)  Change from baseline at Day 7  Change from baseline at Day 90 | 29319 (18255)  -7041 (8893)  -9848 (9611) | 43307 (34140)  -9023 (14731)  -14110 (18296) | -13988 (-28438 to 461)  1982 (-4800 to 8765)  4262 (-4056 to 12580) | 0.258  0.655 |

All data are presented as mean (± SD) Abbreviations of test names are: OTS N1C: Mean Number first choice or the mean of the total number of problems out of 20  solved on first choice OTS M1C : Mean first choice or the total mean number of unique box choices made on each of the 6 problems to find correct solution OTS L1C : Latency first choice or the mean latency time between ball appearance and screen touch for unique box choices made on each of the 6 problems to find correct solution

**: Change from baseline was compared between trial groups with t tests. In addition, a modification of the effect from day 7 to day 90 was tested with an interaction term in a linear regression model with mixed effects and was not found statistically significant.
